# Understanding the course of COVID-19-induced pneumomediastinum

**DOI:** 10.1097/01.JAA.0000794992.99292.48

**Published:** 2021-10-26

**Authors:** Rocco Panico, Jenny Cai, Christopher A. Butts, Jennifer Q. To

**Affiliations:** **Rocco Panico** is lead critical care advanced practice provider at Robert Wood Johnson Barnabas Health-Somerset in Somerville, N.J. **Jenny Cai** is an assistant professor at Rutgers-Robert Wood Johnson Medical School in New Brunswick, N.J. **Christopher A. Butts** is an assistant professor of surgery at Drexel University School of Medicine's Reading, Pa., campus; a trauma/acute care surgeon at Reading Hospital; a clinical assistant professor of surgery at Rutgers-Robert Wood Johnson Medical School; and a clinical instructor in general surgery at Philadelphia (Pa.) College of Osteopathic Medicine. **Jennifer Q. To** is a clinical adjunct assistant professor of surgery at the Lewis Katz School of Medicine at Temple University in Philadelphia, Pa., and practices at St Luke's University Health Network in Bethlehem, Pa. The authors have disclosed no potential conflicts of interest, financial or otherwise.

**Keywords:** COVID-19, coronavirus, pandemic, pneumothorax, pneumomediastinum, imaging

## Abstract

Since its discovery, COVID-19 has infected nearly 112 million people and caused about 2.5 millions deaths worldwide. Our understanding of the clinical presentation and complications of COVID-19 is still evolving. Bilateral pulmonary ground-glass opacities on imaging have become characteristic in the diagnosis of COVID-19, but pneumomediastinum has now also been reported in some patients with COVID-19. Reports on the overall prognosis for these patients are conflicting and little information exists regarding long-term complications. This article describes the clinical course of a patient who did not need mechanical ventilation but developed spontaneous pneumomediastinum.

## CASE

A 60-year-old man presented to his primary care physician with hypoxia and shortness of breath.

### History

**FIGURE 1.**
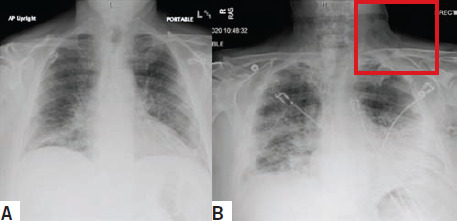
Admission chest radiograph (A) showing bilateral infiltrates. Chest radiograph on hospital day 21, revealing subcutaneous emphysema and pneumothorax at the left lung apex (B, box).

The patient had a history of hypertension, type 2 diabetes, and hypercholesterolemia. On evaluation and workup, he was found to be positive for COVID-19, so he was sent to the ED, where he was found to be febrile (38.7° C [101.7° F]) and hypoxic (SpO_2_ of 87% on room air). Admission laboratory results were significant for a white blood cell (WBC) count of 8,440 cells/mm^3^ (normal range, 4,000 to 10,000 cells/mm^3^), fibrinogen of 556.8 mg/dL (normal range, 200 to 437 mg/dL), lactate dehydrogenase (LDH) of 426 U/L (normal range, 125 to 220 U/L), ferritin of 1,663 ng/mL (normal range, 20 to 175 ng/mL), and procalcitonin of 0.19 ng/mL (normal range, 0.1 ng/mL or less). An admission chest radiograph was notable for bilateral infiltrates consistent with COVID pneumonia (Figure 1A).

**FIGURE 2.**
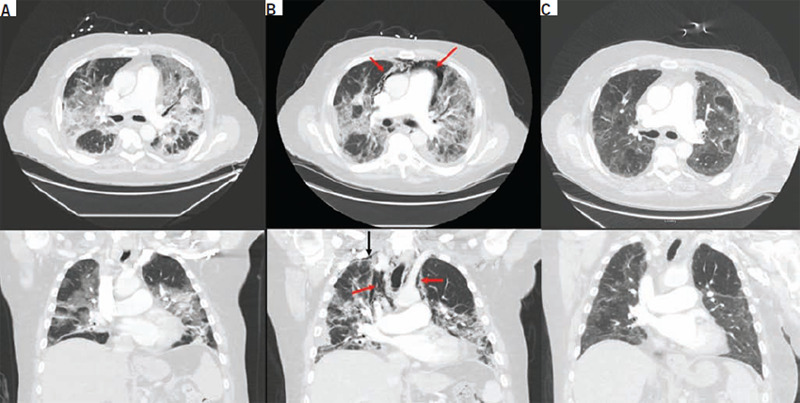
The patient's chest CT (top row, axial views; bottom row, coronal views) on hospital day 11 (A), revealing bilateral infiltrates consistent with COVID-19 pneumonia. Chest CT on hospital day 21 (B), showing pneumomediastinum (red arrows) and a small apical right pneumothorax (black arrow) and bilateral infiltrates. At 12-week follow-up (C), the bilateral infiltrates and pneumomediastinum have resolved.

The patient was admitted to a medical COVID unit, placed on 3 L/min of supplemental oxygen via nasal cannula, treated with intermittent self-proning, and anticoagulated with 0.5 mg/kg enoxaparin twice daily consistent with our hospital's COVID protocol. Convalescent plasma also was given. After experiencing worsening hypoxia on hospital day 14, the patient was transitioned to a high-flow nasal cannula and transferred to the ICU. CT angiography of the chest before ICU admission showed bilateral infiltrates, no pneumothorax or subcutaneous emphysema, and was negative for pulmonary embolus (Figure 2A). While in the ICU, the patient never required intubation, positive-pressure ventilation, or invasive procedures. Despite this, a routine chest radiograph on hospital day 21 demonstrated new subcutaneous emphysema of the left chest and neck (Figure 1B).

Given his continued clinical improvement, the decision was made to monitor him clinically with serial chest radiographs. On hospital day 26, his chest radiograph showed a new right-sided pneumothorax and resolving left-sided subcutaneous emphysema. A chest CT with IV contrast was performed and revealed subcutaneous emphysema, a new small right pneumothorax and significant pneumomediastinum (Figure 2B). Because the patient appeared asymptomatic, he was treated conservatively and monitored closely. His respiratory status continued to improve, and on hospital day 30, he was discharged to a subacute rehabilitation facility on corticosteroid therapy.

**Box 1:**
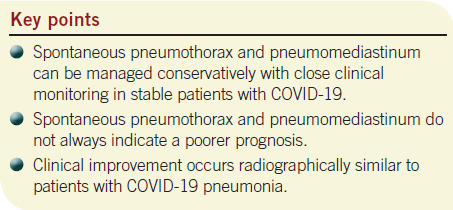
No caption available.

On follow-up evaluation at 6 weeks, a chest radiograph revealed resolving bilateral infiltrates. CT of the chest at 12 weeks showed resolution of pneumomediastinum, improvement of ground-glass opacities, but revealed residual fibrotic changes (Figure 2C).

## DISCUSSION

Although the understanding of the clinical presentation and complications of COVID-19 is still evolving, pneumothorax and pneumomediastinum have been described several times in the literature as a complication of this disease, both in spontaneously breathing patients and those requiring mechanical ventilation.^1^

Spontaneous pneumothorax and pneumomediastinum have been described as complications in previous coronavirus infections, such as SARS-CoV-1 and Middle East respiratory syndrome-related coronavirus infection (MERS).^2^ Patients with neutrophilia, severe lung injury, and a prolonged clinical course were more likely to have a pneumothorax and pneumomediastinum.^2^,^3^ Pneumothorax and pneumomediastinum also were reported to be a marker for poor prognosis.^1^-^3^

Because pneumothorax and pneumomediastinum are known complications of positive-pressure ventilation, some studies suggest that cyst formation in patients with COVID-19 is a potential cause of pneumothorax and pneumomediastinum in the absence of positive-pressure ventilation.^4^,^5^ Cysts progress to bullae in the diseased lung, and can rupture, releasing air into the pleural cavity and mediastinum.^4^,^5^ Increased alveolar pressure and diffuse alveolar injury in patients with COVID-19 pneumonia may result in alveolar rupture, causing pneumothorax and pneumomediastinum.^6^ Excessive coughing could be an example of this mechanism. Coughing leads to increased intrathoracic pressure in COVID-19-damaged alveoli, resulting in alveolar rupture.^7^ Another study has suggested that elevated inflammatory markers in patients with COVID-19 are associated with developing spontaneous pneumothorax.^8^,^9^ Elevated cytokines have been shown to correlate with severity of lung injury in COVID-19.^8^,^9^

Current studies involving patients with COVID-19 and pneumomediastinum have conflicting reports of patients' overall prognosis. Although some studies indicate a poorer prognosis, other studies present a fairly indolent course.^6^,^10^-^12^ Much of the literature on spontaneous pneumothorax and pneumomediastinum consists of single case reports, but a recent multicenter case series sought to examine the clinical characteristics and to prognosticate outcomes in patients with COVID-19 who develop this pathology.^1^ The study concluded that pneumothorax and pneumomediastinum cannot be used independently as a prognostic indicator in patients with COVID-19.^1^ Although this case series consists of the largest number of patients to date, no consensus exists on the management or treatment options for patients with pneumothorax or pneumomediastinum and COVID-19, nor does the study provide follow-up data for these patients.^1^

The case patient was a nonsmoker with no previous history of pulmonary disease. He never required mechanical ventilation nor did he have any evidence of cysts or bullae on imaging. We believe that his pneumomediastinum was secondary to excessive bouts of coughing. Although some reports imply a poorer prognosis or a worse clinical course, our patient improved clinically with conservative management, and we were able to follow up with him after discharge.

Little has been reported on these patients after the acute presentation. Our case report is unique in that our patient was followed for 3 months after hospital discharge. Consistent with ongoing research, this patient's follow-up imaging showed persistent bilateral infiltrates at 6 weeks and improvement of ground-glass opacities at 12 weeks.^13^ His clinical course may indicate that pneumomediastinum does not invariably signify a poorer prognosis and that patients may not exhibit any long-term complications.

## CONCLUSION

Understanding the possible manifestations and examination findings of COVID-19 can help clinicians provide better treatment for these patients. Although COVID-19-induced pneumomediastinum can cause life-threatening decompensation, it can be managed conservatively with successful outcomes. Additional studies are necessary to understand the pathogenesis, risk factors, management, and long-term outcomes of pneumomediastinum and its use as a prognostic indicator in patients with COVID-19.
